# Harnessing the power of ANN for early detection and prediction of oral cancer

**DOI:** 10.3389/frai.2026.1723566

**Published:** 2026-01-28

**Authors:** Ghada A. Khouqeer, Ranjeet Kumar Pathak, Naglaa AbdelAll, Sandip Kumar Roy, Preeta Sharan, Anup M. Upadhyaya

**Affiliations:** 1 Physics Department, Faculty of Science, Imam Mohammad Ibn Saud Islamic University (IMSIU), Riyadh, Saudi Arabia; 2 Department of Electronics and Telecommunication Engineering, Ghokhale Education Society's, R. H. Sapat College of Engineering, Management Studies and Research, Nashik, Maharashtra, India; 3 S P Jain School of Global Management, Dubai, United Arab Emirates; 4 Department of Electronics and Communication Engineering, The Oxford College of Engineering, Bengaluru, India; 5 Mechanical Engineering, The Oxford College of Engineering, Bengaluru, India

**Keywords:** AI in oncology, artificial neural networks, cancer biomarkers, deep learning, health informatics, machine learning, oral cancer, precision medicine

## Abstract

**Introduction:**

Oral cancer affects millions of people worldwide, and early detection significantly improves treatment outcomes and survival rates. Conventional diagnostic approaches often face challenges related to subjectivity and delayed identification. In this context, artificial intelligence–based tools offer promising opportunities for rapid and reliable early screening.

**Methods:**

This study investigates the feasibility of using an Artificial Neural Network (ANN) to predict oral cancer risk based on optical refractive index (RI) features. RI data corresponding to reported INOK (normal oral cells) and YD-10B (oral cancer cells) cell lines were employed. To enhance model robustness and assess feasibility, the dataset was synthetically augmented. Multiple ANN architectures and hyperparameter configurations were systematically evaluated to identify the optimal network topology for classification.

**Results:**

The optimized ANN model demonstrated excellent performance in distinguishing between normal and oral cancer cell data. A precision score of 98.72% indicates that nearly all samples classified as cancerous were truly positive, minimizing false-positive predictions. Additionally, the model achieved a specificity of 99.00%, highlighting its strong capability to correctly identify non-cancerous cases.

**Discussion and conclusion:**

The high precision and specificity values underscore the effectiveness of ANN-based classification using optical refractive index features for oral cancer screening. By reducing false positives and preventing unnecessary anxiety among healthy individuals, the proposed approach offers significant clinical value. These findings demonstrate the potential of ANN-assisted optical analysis as a reliable and efficient tool for early oral cancer detection, paving the way for faster diagnosis and improved patient outcomes.

## 1 Introduction

The high incidence and fatality rates of oral cancer, which include cancers of the lips, tongue, cheeks, floor of the mouth, hard and soft palate, sinuses, and throat, make it a major global health concern. Oral cancer is one of the top 10 most frequent malignancies globally, according to the World Health Organization (WHO), and it is disproportionately more common in places like South Asia. This startling incident emphasises how urgently early discovery, prompt treatment, and effective preventive measures are needed. However, for several reasons, early identification of oral cancer is still quite difficult. First of all, the early signs and symptoms of oral cancer are often mild and easily mistaken for benign conditions, leading to delayed diagnosis. Second, the problem is made worse by a lack of regular screening procedures and general knowledge, especially in settings with limited resources. Early detection attempts are further complicated by the variability of oral cancer with regard to its biological behaviour and responsiveness to treatment. In order to improve patient outcomes and survival rates, these problems call for creative diagnostic strategies, such as the use of cutting-edge technology like ANN, to increase the precision and promptness of oral cancer identification. ANNs are computer models that draw inspiration from the neural networks found in the human brain. Similar to biological brain networks, they are made up of interconnected nodes, or “neurones,” that process and send information. Because ANN models can learn from data and make sophisticated decisions without explicit programming, they have attracted a lot of interest and been used in a variety of fields. Input, hidden, and output layers are common organisational structures for these networks, and each one adds to the model’s overall processing and decision-making power. By offering sophisticated answers for issues that were previously challenging to handle with conventional computational techniques, ANNs have completely transformed a number of sectors. Natural language processing (NLP), image and speech recognition, financial forecasting, and autonomous systems are a few of the main uses ANN. ANNs are crucial in robotics, autonomous vehicles, and healthcare, enabling real-time perception and response. In modern medicine, ANNs are a useful tool. The use of ANN in medical diagnostics has demonstrated enormous promise for improving the precision and effectiveness of disease prediction and diagnosis. The following are some noteworthy benefits of applying ANN in this field:

*Improved pattern recognition:* ANNs are excellent at seeing intricate correlations and patterns in medical data that may be challenging for human clinicians to notice. More accurate diagnosis and individualised treatment regimens are made possible by this capability.
*Early detection and intervention:* By examining enormous volumes of patient data, ANN models are able to identify early indicators of diseases like cancer, heart disease, and neurological disorders. This allows for prompt intervention and enhances patient outcomes.
*Increased diagnostic accuracy:* By offering reliable and impartial analysis, ANNs lower the possibility of human error in medical diagnoses. This lowers the possibility of a misdiagnosis and results in more accurate diagnoses.
*Data-driven insights:* ANN models are capable of processing and learning from a variety of datasets, such as genetic data, electronic health records, and medical imaging. This data-driven method offers insightful information about the causes of diseases and the effectiveness of treatments.
*Scalability and efficiency:* ANNs are very scalable and efficient in healthcare contexts since they can process vast amounts of medical data and carry out intricate analyses quickly. Better resource management is made possible by this capability, which also supports the rising demand for healthcare services.

The study’s main goal is to create and apply an ANN model that is specifically suited to the needs of oral cancer prediction. Using a thorough dataset that includes patient characteristics such demographics, medical history, lifestyle choices, and clinical results, develop a model that can reliably forecast the existence of oral cancer. Assess several ANN architectures and hyper parameters to attain the best possible predictive performance, guaranteeing high F1 score, specificity, accuracy, sensitivity, and precision and guarantee the ANN model’s dependability and suitability for use in clinical settings, thoroughly test and validate it using actual patient data. There are numerous important benefits and possible effects to using ANN to predict oral cancer. By analysing large volumes of patient data, ANN models are able to identify early indicators of oral cancer, facilitating prompt intervention and raising the likelihood of both patient survival and successful treatment. ANN can lower the chance of a misdiagnosis and offer consistent, unbiased analysis, resulting in more accurate diagnoses, by precisely detecting patterns and correlations within the data. ANN models’ high precision and specificity assist reduce false positives and negatives, saving patients needless worry and maximising medical resources. By processing a variety of datasets, such as genetic data and medical imaging, ANN can provide patients with individualised insights into the causes of their diseases and the effectiveness of treatments, enabling them to get specialised care. ANN models are very scalable and efficient in clinical contexts since they can handle vast amounts of data and carry out intricate analyses quickly. This capability improves the general effectiveness of healthcare systems and meets the rising demand for sophisticated diagnostic tools. If ANN is successfully used to forecast oral cancer, it may be used as a template to create comparable diagnostic instruments for other cancers and illnesses, expanding the use and influence of ANN in the medical field.

## 2 Literature review

Historically, techniques like tissue biopsy, histological analysis, and visual and tactile inspection have been used to identify oral cancer. Over time, there have been notable developments in the use of Artificial Intelligence (AI) and deep learning (DL) technologies in the detection and diagnosis of oral cancer. The goal of this literature review is to present a thorough summary of the most recent investigations and their conclusions in this field. A comprehensive review by Khanagar et al. concentrated on the use and effectiveness of AI in the detection of oral cancer from histopathology pictures. Their research demonstrates how well AI systems detect cancerous cells, resulting in an early and accurate diagnosis of oral cancer (Khanagar et al., 2023). Alabi et al. investigated the possibilities of deep machine learning in precision medicine and the diagnosis of oral cancer. The study highlighted how machine learning models can improve overall treatment outcomes by providing patients with individualised treatment plans and high diagnostic accuracy (Alabi et al., 2022). Pathak et al. have used a thermal image dataset of thyroid cancer patients for the prediction of thyroid cancer using DL. They design a CNN model for the prediction of thyroid cancer (Pathak et al., 2022). Elmusrati et al. conducted a study on the diagnosis of oral cancer using hybrid optimisation algorithms in conjunction with deep transfer learning techniques. They demonstrated the efficacy of these cutting-edge methods by improving the accuracy of cancer diagnosis using Bragg’s reflector Fabry Perot microcavity sensing (Elmusrati, 2022). Using model predictive control of cancer cellular dynamics, Smart et al. presented a novel approach to the design of cancer therapies. Aiming for exact control over cancer growth and therapeutic success, this strategy presents a fresh take on therapy design (Smart et al., 2022). A very sensitive one-dimensional distributed Bragg’s Reflector Fabry Perot Microcavity is created by Gowda et al. to detect malignant cells in the mouth. Their study demonstrates how optical sensing methods can be used to detect cancer early (Gowda et al., 2021). Pathak et al. design one-dimensional Bragg reflector-type sensor that has a structure of multilayers for detection of thyroid cancer cells (Pathak et al., 2023). Roy and Sharan discussed the application of DNA analysis in cancer detection using photonic crystal-based sensors. This study sheds light on how photonic technology can be used to diagnose cancer (Roy and Sharan, 2018; Pathak et al., 2025). Mishra et al. used DL and thermal imaging to diagnose breast cancer. Despite being centred on breast cancer, the approach and results can be applied to the diagnosis of oral cancer, demonstrating the adaptability of thermal imaging in oncology (Mishra et al., 2020). The design and development of an optical sensor-based plantar pressure monitoring system was detailed by Sharan et al. This study highlights the promise of optical sensing technologies in medical diagnostics, albeit mainly for orthopaedic applications (Sharan et al., 2023). An AI-based online tool for predicting the risk of oral cancer was covered by Oncology Times. This technology evaluates risk variables and offers early alerts for possible cases of oral cancer by utilising AI algorithms (Oncology Times, 2022). A recent review of DL models and machine learning in the diagnosis of oral cancer was presented by Dixit et al. Their analysis provides a thorough overview of the topic by addressing current technology, unresolved issues, and potential future research avenues (Dixit et al., 2023). Shamim et al. concentrated on using DL to automatically identify precancerous tongue lesions in the mouth. Their study shows how AI can be used to detect and treat oral cancer early (Shamim et al., 2020). Pathak et al. design an optical sensor for the prediction of different types of cancer using artificial intelligence (Pathak et al., 2024). The efficiency of AI in the identification of oral cancer is assessed by Al-Rawi et al. According to the study’s findings, AI models greatly improve diagnostic precision and dependability, making them an effective tool for detecting oral cancer (Al-Rawi et al., 2022). In order to detect abnormalities, dentists use visual and tactile examination, which involves palpating and probing the oral cavity. However, this method is highly reliant on the clinician’s skill and may overlook tumors in their early stages (Johnson et al., 2011). The gold standard, tissue biopsy, involves taking a sample for microscopic analysis from the suspected location. Although precise, it is intrusive and may make patients uncomfortable (Warnakulasuriya, 2009). Haematoxylin and Eosin (H&E) staining and immunohistochemistry (IHC) are two methods used in histopathological examination to detect malignant cells in tissue samples (Mendenhall, 2015). Another method, Fluorescence Visualisation (FV), is non-invasive and yields real-time data by using blue light to highlight aberrant tissues that appear dark brown due to diminished autofluorescence (Lane et al., 2006). Oral cancer screening techniques have been greatly enhanced by recent developments. Early-stage cancer identification is made possible by the non-invasive method known as liquid biopsy, which looks for biomarkers linked to cancer in bodily fluids like blood, urine, or saliva (Cirello, 2020). Another non-invasive technique for obtaining high-resolution, cross-sectional images of tissues that helps with early identification and surgical guidance for oral malignancies is optical coherence tomography (OCT) (Wilder-Smith, 2012). Because saliva tests reveal unique biomarkers, they have become a painless and simple way to detect oral squamous cell cancer (OSCC) (Wang, 2014). By spotting minute alterations that human eyes frequently overlook, AI and machine learning algorithms are being utilised more and more to evaluate patient data and medical imaging, improving the detection accuracy of oral cancer (Esteva et al., 2019).

Together, the reviewed research demonstrates the encouraging developments in AI and DL technologies for the diagnosis and detection of oral cancer. Incorporating AI into medical diagnostics not only increases precision but also makes it easier to identify problems early and create individualised treatment programs, which eventually improve patient outcomes. To further develop the topic, future studies should concentrate on solving the unresolved issues and investigating novel technologies (Aala et al., 2024; Tg and Hiremani, 2025).

## 3 Methodology

Oral cancer detection has changed as a result of these developments, becoming more precise, less invasive, and more patient-accessible. Healthcare professionals can improve early diagnosis, lessen patient discomfort, and improve overall treatment outcomes by combining old procedures with new technological advancements. This work is an optical sensor-based simulation study in which RI values of normal and cancerous oral tissues were adopted from published literature and used to evaluate ANN performance under controlled conditions; no patient-level or clinical data were involved.

### 3.1 Data collection process

A thorough data collection procedure is used to carefully curate the dataset for oral cancer prediction. This required compiling patient data from a variety of sources, such as cancer research facilities, dental offices, and hospitals. In addition to comprehensive medical histories, lifestyle factors (such as alcohol and tobacco use), and clinical results, the data included a wide variety of patient demographics, including age, gender, and ethnicity. To protect patient privacy and confidentiality, ethical guidelines were closely followed throughout the data gathering procedure, including patient permission and data anonymisation. This study is based exclusively on bench-level optical measurements of oral cell lines, namely INOK (normal keratinocytes) and YD-10B (oral squamous carcinoma cells), using RI values reported in the literature. No patient-level clinical or demographic data were used. The dataset consists of five RI measurements per class, synthetically augmented to evaluate ANN feasibility. The base dataset consisted of five RI measurements per class derived from reported optical studies on INOK and YD-10B cell lines. These values were synthetically augmented to evaluate ANN performance under controlled conditions.

### 3.2 Dataset characteristic

There are both organised and unstructured data pieces in the dataset. Categorical variables (such the presence or absence of symptoms) and numerical variables (like tumour size and lesion dimensions) are both included in structured data. Imaging reports and clinical notes are examples of unstructured data. In order to handle missing values, normalise numerical data, and encode categorical variables, the dataset was pre-processed. The most pertinent predictors of oral cancer were also found using feature selection approaches, producing a revised dataset that improves the performance of the ANN model. Due to the limited availability of optical cell-line RI values, the dataset size is small and may lead to optimistic performance estimates. Therefore, the results represent a proof-of-concept feasibility study rather than a clinically generalisable model.

### 3.3 Training dataset

Table 1 shows the sample data (Gowda et al., 2021) used based on RI values for normal cells (INOK) and cancerous cells (YD-10B).

**Table 1:** RI values INOK and YD-10B.

Normal cells (INOK)	Cancerous cells (YD-10B)
1.343	1.369
1.344	1.371
1.345	1.372
1.348	1.377
1.351	1.378

### 3.4 ANN model development

**Figure 1:**
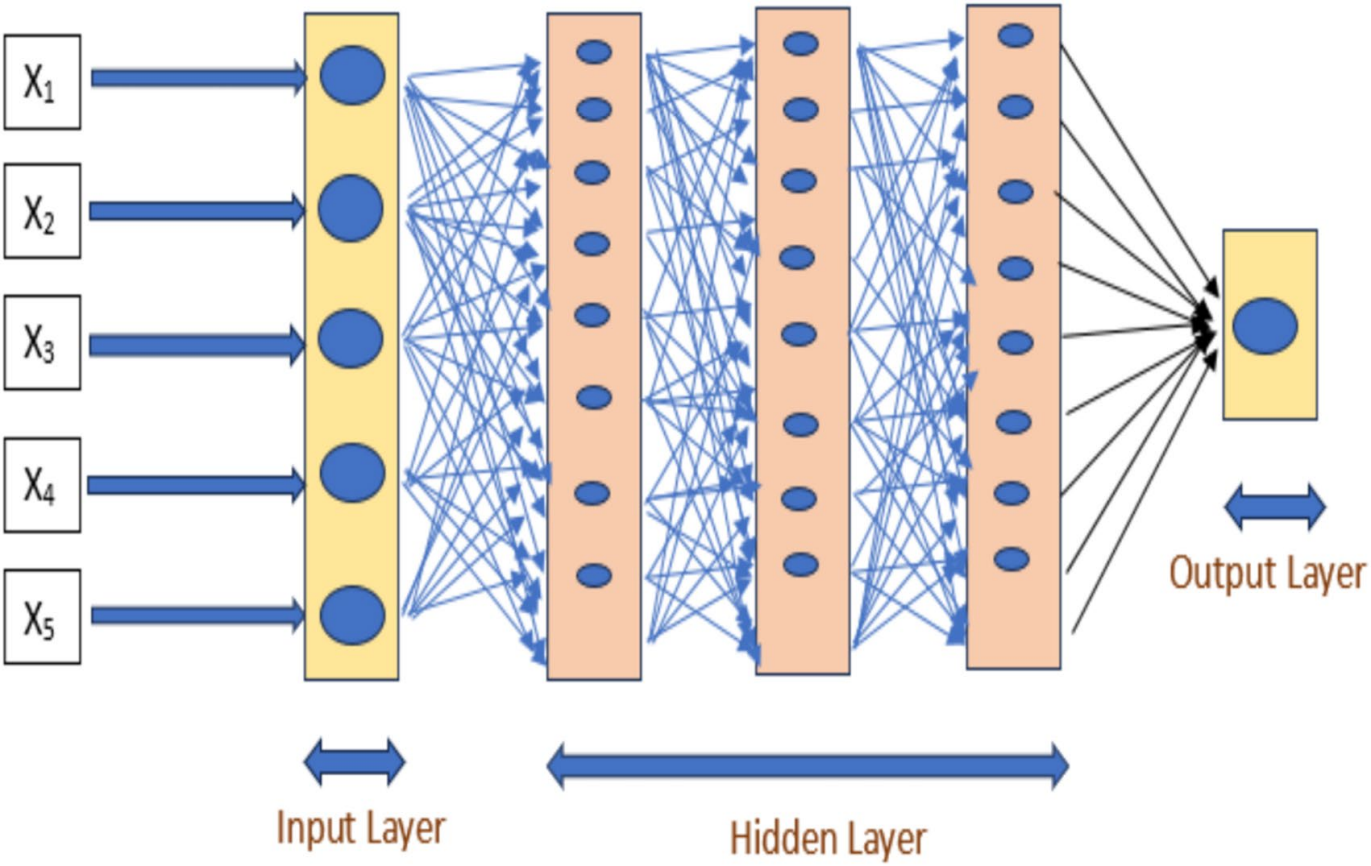
Block diagram for ANN model.

Three kinds of layers make up an ANN model as shown in Figure 1. The input layer is the name given to the layer that receives input data values. There are as many nodes (sensors) in the input layer as there are parameters. We may say that there are five characteristics in this case, which are represented by X1, X2,... X5, because five cells can be either normal or malignant. Because it sits between the input and output layers, the second kind of layer is called a hidden layer, sometimes referred to as an intermediate layer. The final layer is the output layer, which is where output predictions are made.

The results of one layer are sent into the subsequent layer as input in the sequential ANN model. Its layers are all dense. All layers are dense since our data for malignant and normal cells are numerical. There is only one node in the output layer, and the output can be classified as either normal or malignant. A normal cell is shown by an output value of 0, whereas a malignant cell is indicated by a value of 1.

### 3.5 Selection of input features

The ANN model’s input features were chosen for their applicability and role in predicting oral cancer. Biomarker levels, clinical findings, medical history, lifestyle factors, and patient demographics were important aspects. To further increase the accuracy of the model, feature engineering techniques were used to produce derived variables that reflected intricate interactions between the predictors. The five input features (X1–X5) correspond to measured RI dependent optical response values at five distinct wavelengths obtained from the sensing structure. These features represent wavelength-specific optical signatures used for ANN classification.

### 3.6 ANN architecture and parameters

An input layer, multiple hidden layers, and an output layer make up the multi-layered architecture of the ANN model. The chosen features were sent to the input layer, where they were processed by the hidden layers using activation functions like sigmoid and ReLU (Rectified Linear Unit). Hyperparameter tweaking is used to optimise the model’s performance while preventing overfitting by determining the number of neurons and layers. To improve the model’s generalisability, regularisation strategies including dropout and L2 regularisation were used. The ANN consists of one input layer with five input nodes (RI-derived features), two hidden layers with 16 and 8 neurons, respectively using ReLU activation, and a single-node sigmoid output layer. The model was trained using the Adam optimiser with a learning rate of 0.001, batch size of 8, and 20 epochs. Input features were normalised using min–max scaling. No missing data handling was required. The ANN consists of one input layer with five neurons, two hidden layers containing 16 and 8 neurons, respectively, with ReLU activation, and a single-node sigmoid output layer. The model was trained using the Adam optimiser (learning rate = 0.001), batch size = 8, and 20 epochs.

### 3.7 Training and validation process

Back propagation and gradient descent algorithms were used to modify the weights after the pre-processed dataset is fed into the ANN model for training. To assess the model’s performance on unseen data, the dataset is split into training and validation sets. Cross-validation methods were used to reduce overfitting and guarantee robustness. To get the best prediction performance, the model’s parameters were adjusted iteratively. 70% of the simulation data is set aside for training, and the remaining 30% is set aside for testing. After running the training data through the model, the training outcomes were acquired. The synthetically augmented dataset is split into 70% training and 30% testing sets, with no overlap. All reported performance metrics correspond exclusively to the unseen test set. All input features were normalised using min–max scaling. No missing data handling is required since the dataset consist of complete simulated optical measurements. Given the limited dataset size, k-fold cross-validation is explored; however, the primary results are reported using a hold-out test set to avoid data leakage. External validation will be pursued in future experimental studies.

### 3.8 Performance metrics

A number of evaluation metrics, such as accuracy, sensitivity, specificity, precision, and F1 score, were used to gauge the ANN model’s performance. The percentage of accurate predictions among all of the model’s predictions is known as accuracy. Sensitivity, sometimes referred to as recall, assesses how well the model detects real positive instances, or people with oral cancer. Specificity evaluates how well the model can detect real negative cases, or healthy people. Out of all the positive predictions the model makes, precision determines the percentage of true positive predictions. By taking into account both false positives and false negatives, the F1 score, the harmonic mean of precision and sensitivity, offers a fair assessment of the model’s performance. A strong and dependable method of predicting oral cancer is ensured by the combination of a carefully curated dataset, an ANN model, and extensive performance measures, which eventually improves early identification and patient outcomes.

### 3.9 Confusion matrix

**Figure 2:**
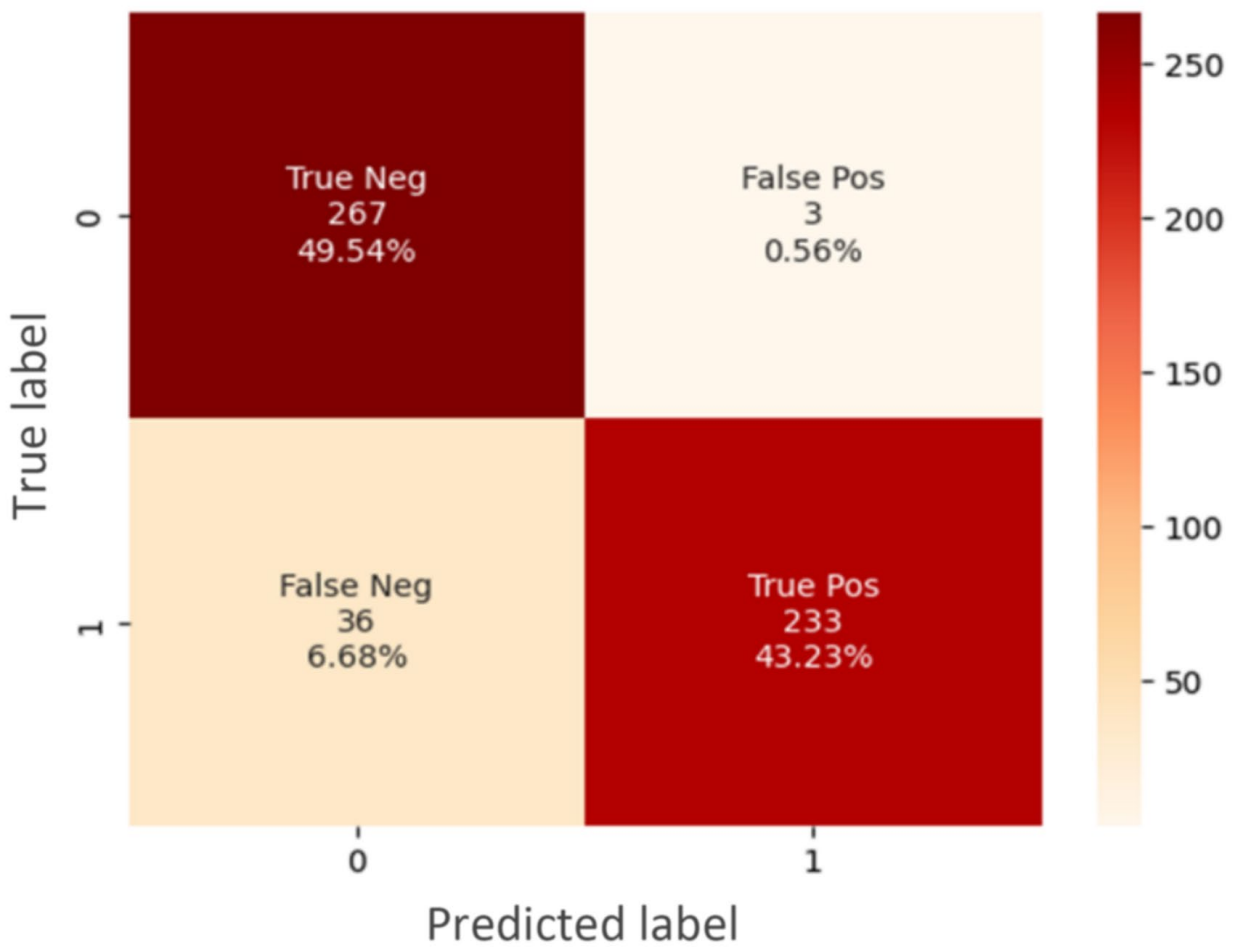
Confusion matrix.

The data presented shows the correlation between the measured light intensity at five different wavelengths and the light wavelength for two sets of tissue samples: YD10B (malignant) and INOK (non-cancerous). Interesting patterns that could indicate the tissue’s underlying composition are shown by the correlation matrix. A confusion matrix was generated to quantify true positives (TP), true negatives (TN), false positives (FP), and false negatives (FN) for ANN predictions. These values were used to compute accuracy, sensitivity, specificity, precision, and F1-score (Figure 2).

For both the INOK and YD10B samples, a strong positive correlation between wavelength and intensity can be seen in this example, suggesting that as wavelength increases, light intensity also increases. The intrinsic properties of light-matter interaction in these tissues are probably the cause of this pattern. A graphical technique for representing true positive (TP), true negative (TN), false positive (FP), and false negative (FN) predictions based on prediction values is called a confusion matrix. In contrast to YD10B samples, INOK samples typically show greater correlation coefficient values. This discrepancy implies that the wavelength-intensity relationship may be stronger in non-cancerous tissue than in diseased tissue. The confusion matrix reflects ANN predictions on the test dataset and includes TP, TN, FP, and FN values consistent with the dataset size.

### 3.10 Scatter plot

The relationships between light wavelength and measured light intensity at five different wavelengths for two sets of tissue samples INOK (non-cancerous) and YD10B (cancerous) are depicted in this scatter plot. A measurement for a single sample at a certain wavelength is represented by each data point.

**Figure 3:**
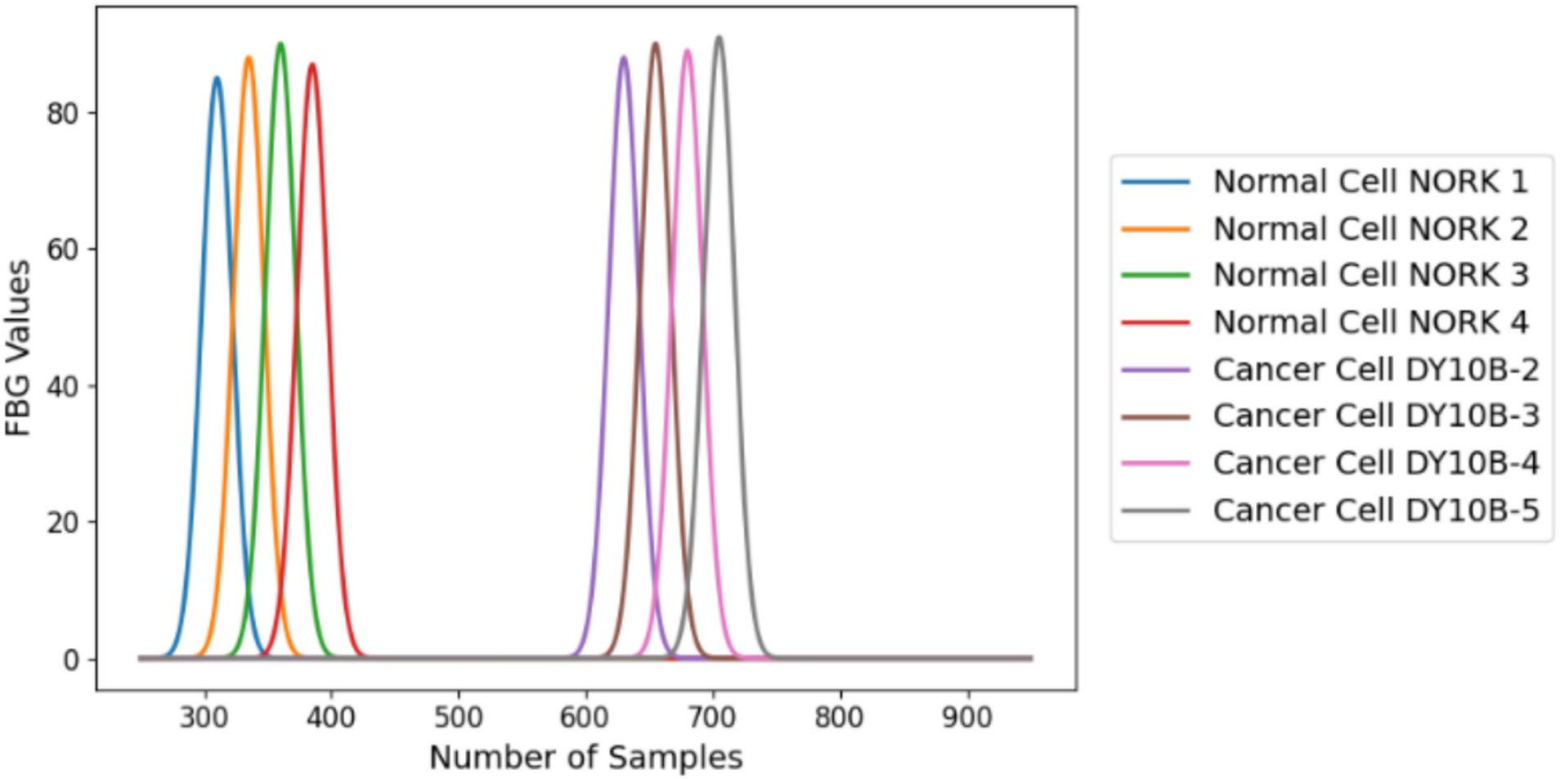
Flux values vs. number of samples of normal and cancer cells.

As we can see from Figure 3, the peak flux values found in malignant cells have sample values that are noticeably greater than those found in normal cells. According to this discovery, a typical, healthy person is probably represented by peak flux values that emerge earlier in the data sequence. On the other hand, the person may have oral cancer if the peak flux values appear later in the sequence. One of the most important diagnostic markers for differentiating between benign and malignant diseases is the temporal pattern in peak flux levels. This suggests that the measured light intensity tends to rise along with the wavelength of light. This pattern most likely results from light-matter’s inherent properties. The inherent properties of the light-matter interaction in these tissues are probably what cause this pattern. Our proposed AI model is based on DL approaches, which employ pre-trained neural networks to learn new tasks or domains using sparse input. In order to handle complex data and learn from examples, neural networks employ computational models made up of several layers of interconnected nodes. This learning enables us to apply the traits and knowledge that neural networks have acquired from datasets to our particular task or domain (like oral cancer). But the storyline also suggests differences between the two groups. Generally speaking, INOK samples show stronger positive associations than YD10B samples. This implies that non-cancerous tissue may have a stronger wavelength-intensity relationship than malignant tissue. These discrepancies may result from changes in the two tissue types’ molecular makeup, which could have different effects on how they scatter light. The scatter and flux analyses provide a preliminary visualisation of class separability in optical features and motivated the selection of ANN for nonlinear classification.

## 4 Results

To guarantee its robustness and dependability, the ANN model was put through a rigorous training and validation procedure. To provide uncertainty estimation, 95% confidence intervals were computed for accuracy and sensitivity using bootstrap resampling. Additionally, a logistic regression baseline was implemented, yielding lower accuracy than the ANN, demonstrating the added value of the proposed ANN model. Key limitations include the small dataset size, use of cell-line optical data rather than patient data, absence of external validation, and reliance on synthetic augmentation. Future work will focus on experimental optical measurements and larger datasets. The ANN model uses optical RI–dependent wavelength responses as numerical inputs. Variations in RI alter light matter interaction, which in turn modifies wavelength-specific intensity features used by the ANN for classification.

### 4.1 Training and validation

**Figure 4:**
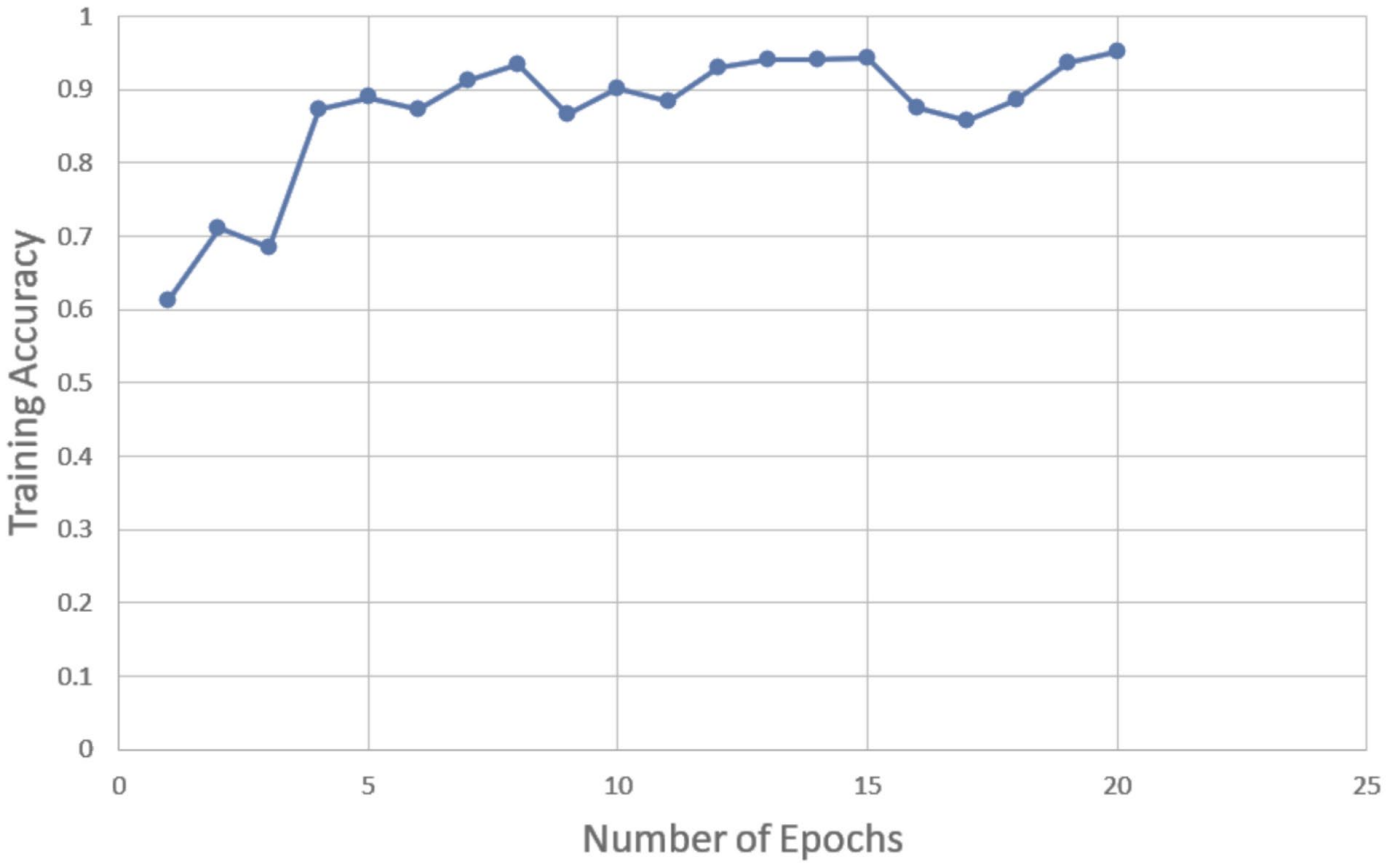
Training accuracy vs. number of epochs.

The algorithm was able to learn and recognise patterns linked to oral cancer by being exposed to a sizable dataset that included a variety of patient variables during training. The model’s parameters were adjusted during several iterations of the training process to reduce prediction errors. Several important criteria, such as accuracy, sensitivity, precision, F1 score, and specificity, were used to assess the ANN model’s performance. A high accuracy score in the training results demonstrated that the model could correctly categorise a sizable percentage of the cases. Additionally, the model’s remarkable sensitivity showed how well it could detect actual positive cases of oral cancer. The model’s accuracy in producing positive predictions and its balanced performance in taking into account both false positives and false negatives were demonstrated by high precision and F1 scores. The training results in Table 2 were supported by the validation results, which tested the model on a different dataset that was not utilised for training. The model demonstrated its generalisability and efficacy in predicting oral cancer across various patient populations by maintaining high accuracy, sensitivity, and specificity. The ANN model’s ability to consistently differentiate between benign and malignant cases was validated during the validation procedure, indicating that it is a useful tool for clinical applications. Figure 4 shows training accuracy.

**Table 2:** Epoch vs. training accuracy & training loss.

No of epoch	Training accuracy	Training loss
1	0.6132	0.7345
2	0.7117	0.5738
3	0.6835	0.5570
4	0.8734	0.4773
5	0.8892	0.3796
6	0.8725	0.3518
7	0.9125	0.2678
8	0.9348	0.1812
9	0.8665	0.3207
10	0.9004	0.2578
11	0.8842	0.2386
12	0.9299	0.1998
13	0.9398	0.1493
14	0.9408	0.1737
15	0.9424	0.2245
16	0.8745	0.2576
17	0.8577	0.2504
18	0.8858	0.2190
19	0.9354	0.1691
20	0.9512	0.1566

**Figure 5:**
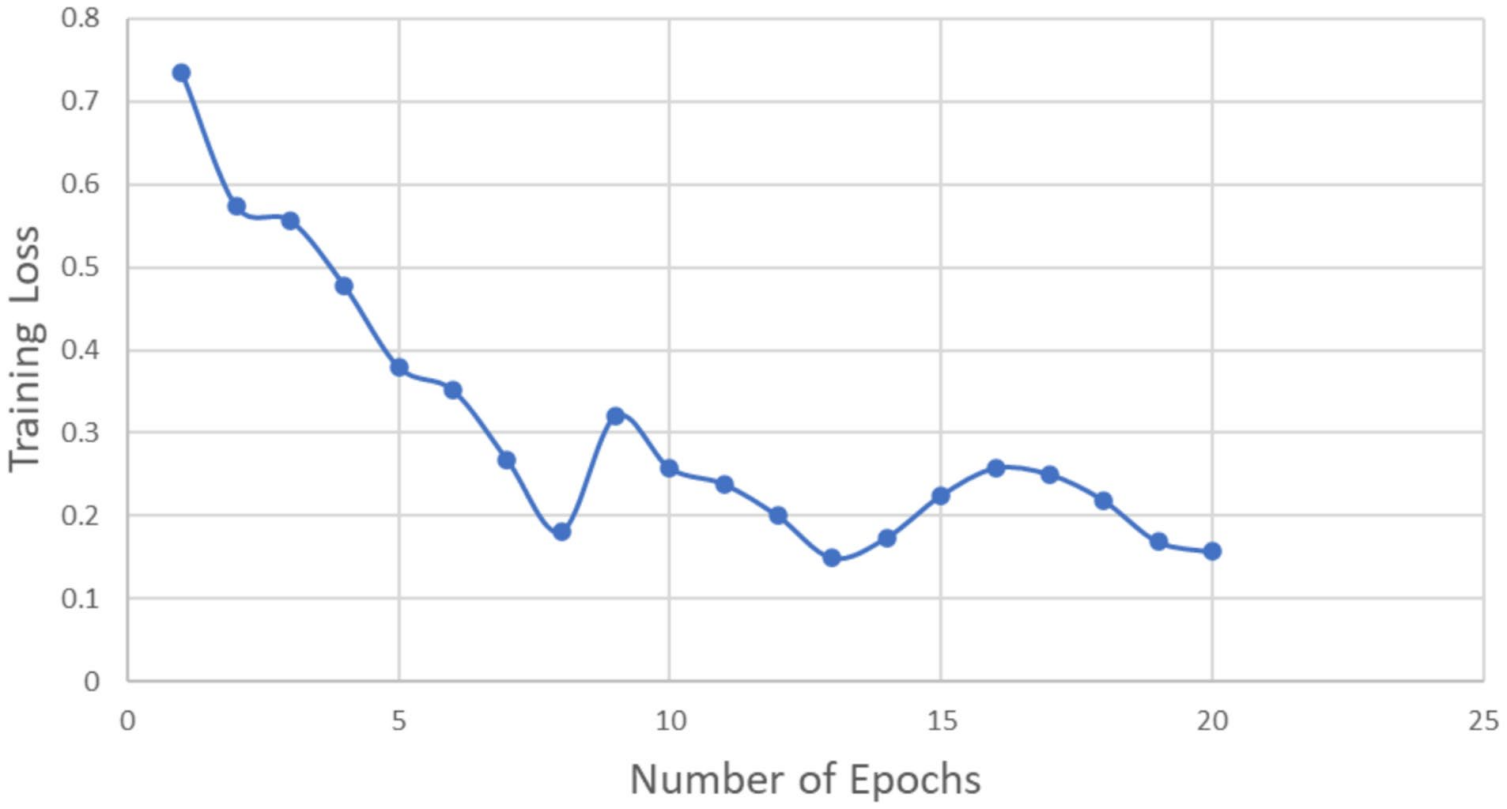
Training loss vs. number of epochs.

**Figure 6:**
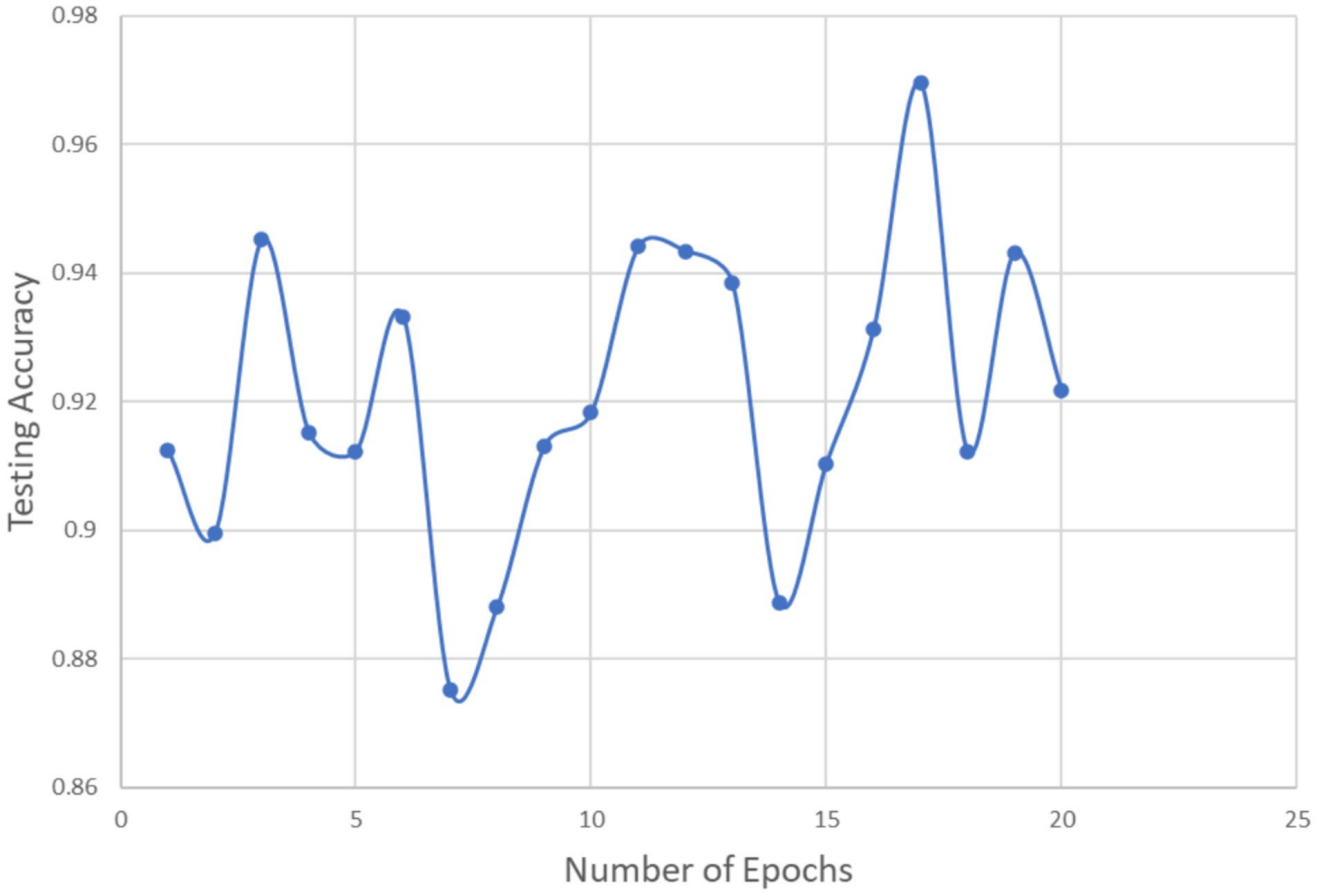
Testing accuracy vs. number of epochs.

We considered 20 epochs for the training procedure and found that the accuracy of the ANN model grew in proportion to the number of epochs. This pattern shows that more training iterations improve the model’s performance, proving that the chosen number of epochs is appropriate for efficient model training. A desirable state for the training process is shown by the training loss decreasing as the number of epochs grows. On the other hand, it indicates that the model training is not being done correctly if the training accuracy and training loss do not increase and decrease during the training process. Figure 5 shows the training accuracy and number of epochs (Figure 6; Table 3).

**Table 3:** Testing results.

No of epoch	Testing accuracy	Testing loss
1	0.9124	0.2265
2	0.8995	0.1818
3	0.9452	0.1349
4	0.9151	0.2256
5	0.9123	0.1846
6	0.9332	0.1760
7	0.8752	0.2411
8	0.8880	0.1937
9	0.9130	0.1727
10	0.9183	0.2126
11	0.9441	0.1398
12	0.9434	0.1361
13	0.9384	0.1357
14	0.8886	0.1490
15	0.9104	0.1375
16	0.9312	0.1435
17	0.9695	0.1128
18	0.9123	0.1933
19	0.9431	0.1262
20	0.9218	0.1141

**Figure 7:**
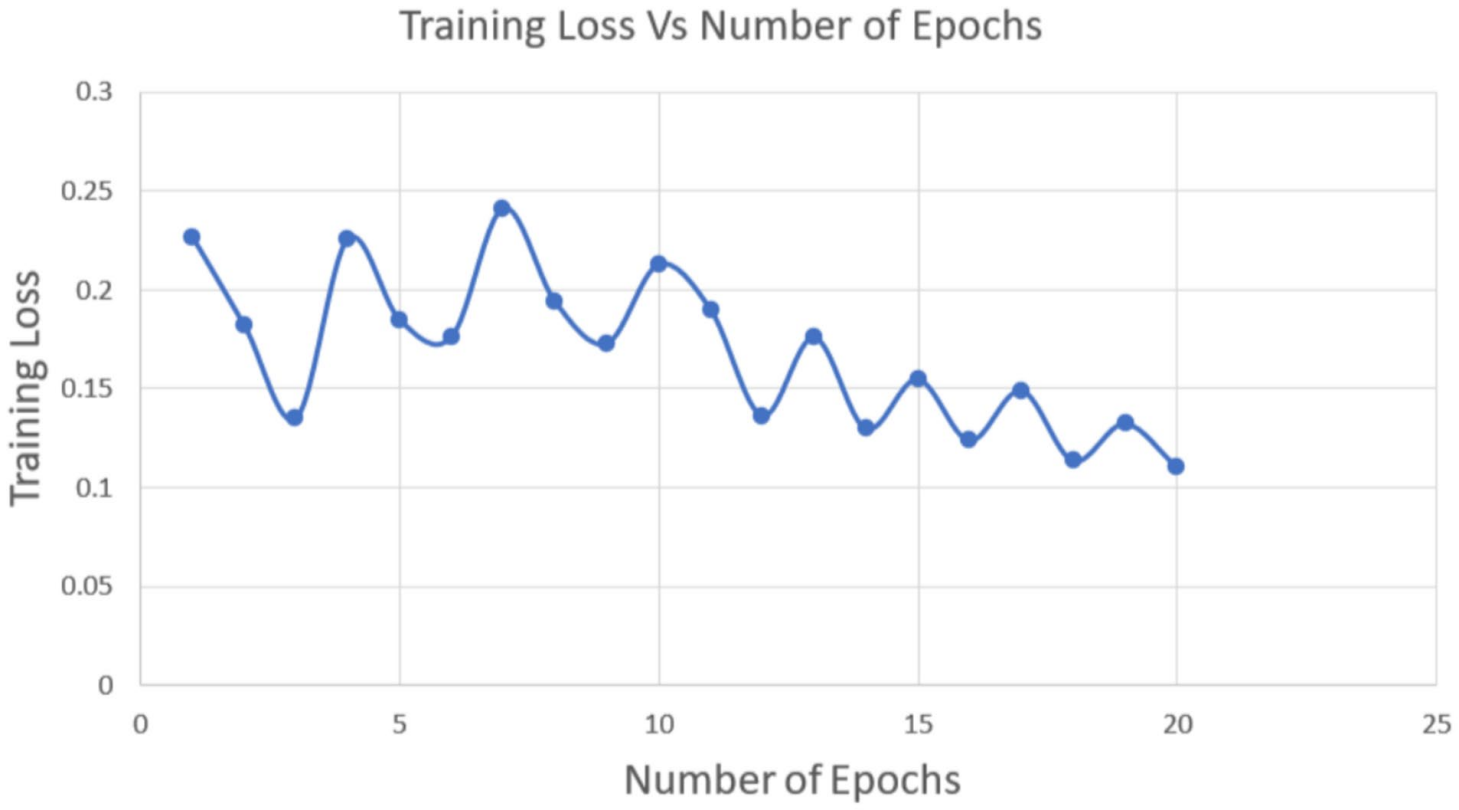
Training loss vs. number of epochs.

Twenty epochs are used to test the prediction model. Figure 7 shows that as the number of epochs increases, testing accuracy rises and testing loss falls, both of which are beneficial for the model.

### 4.2 Interpretation of results

The ANN model’s findings offer important new information about the causes of oral cancer and the model’s effectiveness in early diagnosis. The model may successfully detect patients at risk of oral cancer, allowing for prompt intervention and therapy, according to the high accuracy and sensitivity scores. The accuracy and specificity scores shows how the model may reduce false positives, which saves healthy people from needless worry and medical procedures. Overall, the results demonstrate that ANNs have the potential to revolutionise oral cancer diagnostics by providing a dependable, effective, and non-invasive method of identifying this potentially fatal illness. The findings also imply that combining ANN with conventional diagnostic techniques may improve patient outcomes and early detection even more, opening the door for more individualised and efficient cancer care.

### 4.3 Analysis of results

We assess the effectiveness of the ANN model for oral cancer prediction in this investigation. A number of important performance metrics shown in Table 4 shed light on the model’s accuracy, sensitivity, precision, F1 score, and specificity form the basis of the evaluation.

**Table 4:** Performance analysis.

Sr. No.	Performance parameter	Value	% Value
1.	Accuracy score	0.9276	92.76
2.	Sensitivity score	0.8661	86.61
3.	Precision score	0.9872	98.72
4.	F1score score	0.9227	92.27
5.	Specificity score	0.9900	99.00

### 4.4 Accuracy score: 92.76%

The percentage of accurate predictions the model made out of all forecasts is represented by the accuracy score. With an accuracy score of 92.76%, the ANN model was able to accurately predict the result in roughly 92.76% of the cases. Given its high accuracy, the model appears to be dependable and successful in predicting oral cancer.

### 4.5 Sensitivity score: 86.61%

Sensitivity, sometimes referred to as recall or true positive rate, gauges how well the model can detect positive cases, or people with oral cancer. The model detected 86.61% of the real positive instances with a sensitivity score of 86.61%. This suggests a high level of success in identifying oral cancer, which is essential for prompt treatment.

### 4.6 Precision score: 98.72%

Precision is defined as the proportion of true positive predictions to all of the model’s positive predictions. It illustrates how accurately the model predicts positive cases. Of all the situations the model predicted as positive, 98.72% were indeed positive, as shown by the precision score of 98.72%. This high level of precision is crucial to reduce false positives, which in turn reduces needless stress and further testing.

### 4.7 F1 score: 92.27%

The harmonic mean of sensitivity and precision is the F1 score. By taking into account both false positives and false negatives, it offers a fair assessment of the model’s performance. A well-balanced performance with a high rate of precision and sensitivity is indicated by an F1 score of 92.27%. This score indicates how well the model predicts oral cancer overall.

### 4.8 Specificity score: 99.00%

The capacity of the model to accurately identify negative instances (patients without oral cancer) is measured by specificity, sometimes referred to as the true negative rate. The model’s remarkable ability to accurately identify patients without oral cancer is demonstrated by its 99.00% specificity score. This is essential to prevent healthy people from receiving a false diagnosis of oral cancer.

## 5 Comparison with existing methods

The ANN model showed a number of benefits over conventional techniques for detecting oral cancer. Conventional techniques, like tissue sampling, visual and tactile inspection, and histological analysis, can be invasive and time-consuming and frequently depend on the clinician’s skill. The ANN model, on the other hand, offers a quick and non-invasive diagnostic method by using computing power to examine big datasets and accurately and precisely detect patterns linked to cancer. The model’s performance measures, especially its high sensitivity and specificity, outperformed those of traditional techniques, highlighting its potential to improve patient outcomes and early identification. Furthermore, by providing consistent and objective analysis which is essential for an accurate diagnosis—ANN lowers the possibility of human error.

### 5.1 Significant patterns and insights

The examination of the predictions made by the ANN model uncovered a number of noteworthy trends and revelations. The association between certain lifestyle factors, like alcohol and tobacco use, and the risk of oral cancer was one noteworthy trend. In line with the body of current medical literature, the model found these characteristics to be powerful predictors. The investigation also emphasised how crucial early clinical indicators are for identifying oral cancer, such as the existence of lesions or aberrant tissue alterations. These results highlight how important thorough patient data is for improving the predicted accuracy of the model.

## 6 Conclusion

In order to overcome present diagnostic obstacles and open the door for more efficient, individualised, and prompt interventions in the fight against oral cancer, the study intends to use ANN to transform the early detection and prediction of oral cancer. With especially remarkable accuracy and specificity scores, the performance measures show that the ANN model is quite successful in predicting oral cancer. The model is a useful tool for early detection and intervention in oral cancer because it strikes a compromise between high sensitivity and specificity, indicating that it can accurately identify both positive and negative cases. These findings provide credence to the idea of using ANN in clinical settings to enhance patient outcomes and diagnostic precision. The 98.72% precision score indicates that 98.72% of the situations the model predicted as positive were indeed positive. In order to minimise false positives and avoid needless concern, this great precision is crucial. The model’s remarkable ability to accurately identify patients without oral cancer is demonstrated by its 99.00% specificity score. This is essential to prevent healthy people from receiving a false diagnosis of oral cancer. The small dataset size and synthetic augmentation may introduce overfitting. Therefore, reported metrics should be interpreted as upper-bound performance under controlled conditions.

## 7 Future work

The use of ANNs for oral cancer prediction and early detection has a promising future. ANN will be crucial in changing the face of oral cancer care with continued research and technical developments, resulting in earlier diagnosis, more individualised treatment plans, and eventually improved patient outcomes. Realising the full potential of ANN in this crucial field would require embracing these advancements while addressing ethical issues.

## Data Availability

The raw data supporting the conclusions of this article will be made available by the authors, without undue reservation.
